# Too much of a good thing: compulsive exercise in children and adolescents with eating disorders.

**DOI:** 10.1186/2050-2974-3-S1-O54

**Published:** 2015-11-23

**Authors:** Pam Formby, Hunna J Watson, Anna Hillyard, Kate Martin, Sarah J Egan, Nine Smith-Birch

**Affiliations:** 1 School of Physiotherapy, University of Notre Dame, Fremantle, Australia; 2 Department of Psychiatry, The University of North Carolina at Chapel Hill, Chapel Hill, NC, USA; 3 School of Paediatrics and Child Health, The University of Western Australia, Perth, Australia; 4 School of Psychology and Speech Pathology, Curtin University, Perth, Australia; 5 Complex Pain Service, Princess Margaret Hospital for Children, Perth, Australia; 6 Eating Disorders Program, Specialised Child and Adolescent Mental Health Service, Perth, Australia

## 

Compulsive exercising is the most common compensatory behaviour amongst children and adolescents with eating disorders. The Compulsive Exercise Test is a self-report measure that has been recently developed to assess the cognitive, behavioural and affective features of compulsive exercise. The aim of the current study was to validate the measure using a paediatric eating disorder sample presenting for treatment at a specialist eating disorder service.

Confirmatory factor analysis was conducted and correlations with eating disorder symptoms were examined. The study failed to confirm a factor structure, although there was still clear evidence of the multidimensionality of the measure. CET scores were significantly related to measures of eating pathology, perfectionism and exercise frequency to control shape and weight. This suggests a need for further investigation into the construct of compulsive exercise in clinical populations, given the strong association with eating pathology.

